# A compressive sensing approach for inferring cognitive representations with reverse correlation

**DOI:** 10.3758/s13428-023-02281-4

**Published:** 2023-12-04

**Authors:** Benjamin W. Roop, Benjamin Parrell, Adam C. Lammert

**Affiliations:** 1 Program of Neuroscience, Worcester Polytechnic Institute, Worcester, MA USA; 2 Department of Communication Sciences and Disorders, University of Wisconsin-Madison, Madison, WI USA; 3 Biomedical Engineering Department, Worcester Polytechnic Institute, 100 Institute Rd, Worcester, MA 01609 USA

**Keywords:** Perceptual representations, Reverse correlation, Compressive sensing, Classification images

## Abstract

Uncovering cognitive representations is an elusive goal that is increasingly pursued using the reverse correlation method, wherein human subjects make judgments about ambiguous stimuli. Employing reverse correlation often entails collecting thousands of stimulus-response pairs, which severely limits the breadth of studies that are feasible using the method. Current techniques to improve efficiency bias the outcome. Here we show that this methodological barrier can be diminished using compressive sensing, an advanced signal processing technique designed to improve sampling efficiency. Simulations are performed to demonstrate that compressive sensing can improve the accuracy of reconstructed cognitive representations and dramatically reduce the required number of stimulus-response pairs. Additionally, compressive sensing is used on human subject data from a previous reverse correlation study, demonstrating a dramatic improvement in reconstruction quality. This work concludes by outlining the potential of compressive sensing to improve representation reconstruction throughout the fields of psychology, neuroscience, and beyond.

**Supplementary Information:**

The online version contains supplementary material available at 10.3758/s13428-023-02281-4.

Human perceptual experience is mediated by internal representations of the world around us. Representations bridge the gap between the raw information available from the senses and the abstract and often categorical nature of our perceptual experience by acting as reference patterns for everything from low-level spatial and temporal features (e.g., edges and shapes in the retina image; Hendrickson & Goldstone, 2009) to high-level characteristics of cognitive categories (e.g., faces; Gosselin & Schyns, 2003) or social constructs (e.g., the trustworthiness of a face; Dotsch & Todorov, 2012). Full understanding of perceptual experience therefore depends on the ability to characterize these internal representations, as they encapsulate important abstractions that are necessary to make sense of complex sensory inputs (Fig. 1). While substantial progress has been made toward fully describing lower-level representations (e.g., visual and auditory receptive fields), similar characterization of higher-level cognitive representations has proven to be more elusive. This is largely because, despite their central importance to perceptual experience and behavior (Brinkman et al., 2017), such representations are notoriously difficult to measure (Gosselin & Schyns, 2003; Barth et al., 1999; Hansen et al., 2010; Smith et al., 2012; Varnet et al., 2013).

**Fig. 1:**
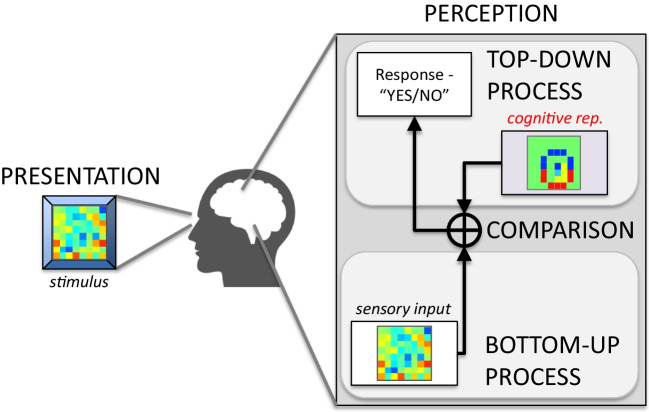
Schematic overview of perception. At its most fundamental level, perception is maintained by a complex cognitive system in the perceiver, involving the combined efforts of bottom-up and top-down processes that bridge the gap between sensory input and cognitive representations. Bottom-up processes extract relevant features from the raw sensory signal, while top-down processes relate incoming features to perceptual categories by comparing against prior expectations in the form of internal, cognitive representations. Such top-down theories are widely posited across multiple domains of human perception (e.g., Gregory, 1997)

One of the more promising methods to overcome this difficulty attempts to uncover higher-level cognitive representations using a standard method for characterizing lower-level neural representations: reverse correlation (De Boer & Kuyper, 1968). Reverse correlation reveals the latent representation in these low-level contexts by eliciting neural responses to richly varying stimuli (e.g., white noise) and regressing the observed responses against the stimuli over many trials. The same method can be utilized in the higher-level context by substituting behavioral responses for neural ones, in much the same way that one can report whether they “see” faces and other familiar shapes in clouds and other ambiguous visual stimuli. Ambiguous stimuli can thus be used to force the internal representations to exert their influence directly on elicited responses. This approach began in the auditory domain, wherein subjects responded on whether they heard a tone within a noise signal (Ahumada and Lovell, 1971). In ensuing years, visual noise was presented to subjects to reconstruct internal representations or “classification images” of target objects (Ahumada, 1996; Eckstein & Ahumada, 2002). In one pioneering study, Gosselin & Schyns (2003) sought to find representations of letters by asking participants to identify a letter “S” in visual noise stimuli; critically, the stimuli were purely noise (and thus did not contain any actual letters), yet reverse correlation based on subject responses revealed a definitive “S” image recovered from each participant. Reverse correlation has subsequently been demonstrated to avoid the shortcomings of other techniques that bias expectations (Chauvin et al., 2005). Other groups have used reverse correlation to characterize the top-down processes of perception to abstract psychological categories, including the trustworthiness in voices (Ponsot et al., 2018), self-image (Moon et al., 2020), and “male” vs. “female” faces (Brinkman et al., 2017), and shown that reverse correlation can quantitatively compare human decision-making to that of computers (Jäkel et al., 2009; Okazawa et al., 2018).

Despite this initial success in certain contexts, reverse correlation has severe limitations that have prevented its wide-spread use for uncovering higher-level internal representations. Most critically, standard applications of reverse correlation require a burdensome number of stimulus-response samples for accurate reconstruction of cognitive representations with an adequate signal-to-noise ratio (Varnet et al., 2013). For example, Gosselin & Schyns’ (2003) experiment required 20,000 trials from each participant (Gosselin & Schyns, 2003); another required 11,400 (Barth et al., 1999). In cognitive and psychological experiments, the large number of trials combined with the long latency for measurable behavioral responses from subjects results in data collection protocols that can last weeks for each individual subject. This inefficiency limits the feasibility of applying reverse correlation to only those experimental protocols where subject participation and motivation can be maintained over long timelines. Even in such cases, the large amount of data required from each participant means the number of participants in a given study is typically very low (usually less than five; Gosselin & Schyns, 2003; Hansen et al., 2010; Smith et al., 2012). This severely limits any insight into the generalizability of existing findings to a broader population.

Existing methods attempt to overcome this fundamental limitation of reverse correlation by constraining the input stimuli to decrease the number of required trials. This can be accomplished, for example, by generating stimuli through adding noise to known examples of some real-world category (e.g., an image of a face) rather than using pure noise, as done in (Moon et al., 2020). This increases the probability that subjects will report the stimulus as belonging to the category of interest but also constrains the results toward representations consistent with the initial example. In order to realize the full impact of reverse correlation in uncovering cognitive representations, it is necessary to reduce the number of trials required without introducing such constraints.

An alternative approach to improving the efficiency of reverse correlation is to impose constraints on the reconstruction process. Adding constraints can be a powerful way to improve reconstruction quality but can also severely limit the richness of the reconstruction in ways prespecified by the constraints themselves. For example, current approaches constrain reconstructions through low-pass filtering (Smith et al., 2012), such as by smoothing with a 2D Gaussian kernel (e.g., as in Gosselin & Schyns, 2003). While this can eliminate unwanted high-frequency noise from the reconstruction, it also presupposes that such high-frequency information does not form part of the underlying representation. For this reason, it is critical to assess the assumptions inherent in any added constraint and to be careful in deciding whether to utilize that constraint (Mineault et al., 2009; Murray, 2011).

One constraint that seems especially appropriate for reconstructing perceptual representations is that of sparsity. Sparsity is the notion that representations are composed of a finite and relatively small set of essential features. Importantly, sparsity is widely considered to be a fundamental principle of organization in perceptual systems at all levels (Olshausen & Field, 2004), with strong empirical and theoretical support (Olshausen & Field, 2004; Hubel & Wiesel, 1968; Olshausen & Field, 1996; Srivastava et al., 2003). Thus, sparsity is a fairly innocuous assumption that is highly likely to preserve the essential aspects and important variation in cognitive representations. This assumption can also be exploited to improve estimation of internal representations (Mineault et al., 2009). Mineault and colleagues incorporated sparsity constraints in a generalized linear model (GLM) to reconstruct visual-domain representations more efficiently, demonstrating an approximately 80% reduction in the number of trials to produce an equivalent quality reconstruction (Mineault et al., 2009).

Recent advances in signal processing have resulted in a proliferation of efficient sampling methods based on the idea of sparsity, collectively known as *compressive sensing* (Mineault et al., 2009; Murray, 2011). To date, however, compressive sensing has not been applied to the reverse correlation paradigm. Here, we explicate the underlying mathematical connections between reverse correlation and compressive sensing and provide a demonstration of the potential for dramatic efficiency improvements using the combined method. We will also show that, unlike previous approaches to incorporating sparsity constraints, it is also possible to obtain reconstructions from compressive sensing analytically, using a closed-form solution that is highly efficient and guaranteed optimal. One such method by Zhang (Zhang et al., 2014) is presented in detail and applied here in a novel way.

Compressive sensing begins with the assumption that signals of interest, *x*, which can include latent cognitive representations (Fig. 2), are sparse (or, in the language of compressive sensing, *compressible*). This means specifically that they can be represented by a small number of functions from an appropriately-selected basis set (*s *= **Ψ**^**T**^x, for basis Ψ and weights *s*). If one assumes that responses, *𝑦*, stem from a process of comparing stimuli to the latent representation (*𝑦 =* Φx, for stimuli Φ), it is possible to estimate the latent representation using only a small number of measurements by acquiring the basis function representation directly (i.e., *𝑦 =* ΦΨ*s*) via sparse optimization approaches to find *s*. In practice, sparse representations may be found even when the chosen basis domain is quite general and incorporates no specific prior knowledge of the signal characteristics (e.g., the discrete cosine transform, wavelet transform). The choice of an appropriate basis function may depend on the domain being interrogated, but here the cosine basis is shown to work adequately.

**Fig. 2:**
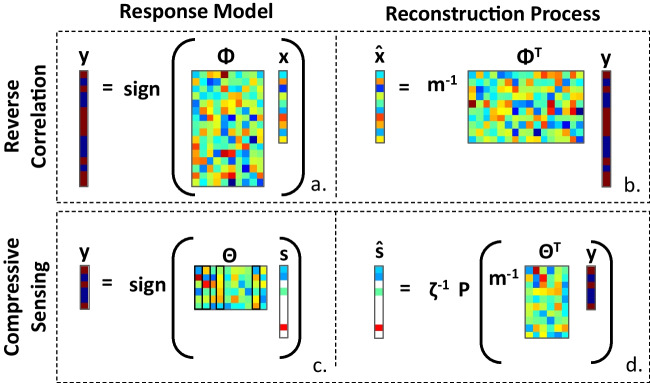
Process of reconstruction in reverse correlation and compressive sensing. (A) In reverse correlation, the vector of subject responses is modeled as resulting from the multiplication of a latent representation vector (*x*) and a stimulus matrix (Φ), where each row of Φ is a presented stimulus. This can be thought of as a similarity calculation between the latent representation and a vector representation of each presented stimulus. (B) An estimate of the latent representation ($\hat{\mathrm{\it x}}$) is then reconstructed by regressing responses against the stimuli. (C) In compressive sensing, the vector of subject responses is modeled as resulting from the multiplication of a sparse latent representation vector (s) and a compressive sensing matrix (Θ). The compressive sensing matrix is formed by multiplying a matrix of basis functions by the stimulus matrix (Θ=ΦΨ), which amounts to a similarity calculation between the stimuli and the known basis functions. (D) An estimate of the sparse latent representation ($\hat{\it s}$) is then reconstructed by regressing the responses against the compressive sensing matrix, soft-thresholding the resulting regression coefficients, and then normalizing by ζ = ||P(m^-1^Θ^T^*y*)||, where m is the dimensionality of the stimulus vector and P is a soft thresholding function. The full representation estimate ($\hat{\mathrm{\it x}}$) can be subsequently calculated using the estimated sparse representation and the known basis functions ($\hat{\mathrm{\it x}}$ = $\Psi \hat{\mathrm{\it s}}$). Note that the response vector y in compressive sensing is generally assumed to contain many fewer entries than in reverse correlation, and without sacrificing reconstruction accuracy

A critical point for the applicability of compressive sensing for reverse correlation is that it has been demonstrated both theoretically (Tropp et al., 2009; Yotsukura et al., 2014) and empirically (Mineault et al., 2009) that using random (e.g., white noise) stimuli to elicit responses is a highly effective way to ensure accurate reconstruction of latent representations within the compressive sensing framework. Moreover, a substantial portion of the literature on compressive sensing has focused on inferring representations from binary responses (e.g., yes-no), a variation of classical compressive sensing called 1-bit compressive sensing (Zhang et al., 2014; Boufounos & Baraniuk, 2008; Jacques et al., 2013). That is, 1-bit compressive sensing maps directly onto the most common (i.e., “yes-no” response) reverse correlation paradigm. This opens the door to potentially providing all the efficiency benefits of compressive sensing to the reverse correlation paradigm.

To this end, computational simulations are used here to model reverse correlation in subject responses to visual stimulation. Internal representations are reconstructed based on these simulated responses using either the traditional reverse correlation approach, the sparse GLM approach used by Mineault (2009), or compressive sensing, and compressive sensing improves both the efficiency of the sampling process and the quality of the reconstructions. Finally, compressive sensing is applied to human subject response data from a prior reverse correlation study (Smith et al., 2012), again demonstrating the ability of compressive sensing to enhance reverse correlation experiments.

## Method

### Modeling subject responses

The general model of subject response behavior underlying reverse correlation experiments is that responses (*y*) are a result of linear comparisons between a latent representation (*x*) and a collection of random stimuli (Φ):

1 $$y=\operatorname{sign}\left(\Phi x\right),$$

where y is a l-by-1 vector of responses, *x* is a m-by-1 representation, and Φ is a l-by-m "measurement matrix". l refers to the number of data points, and m refers to the number of stimulus dimensions. Note that expected yes/no responses from subjects are coded numerically as 1 (yes) and 0 (no) in this formulation. This idealized model does not account for noise that is likely to be inherent in the human subject response process. Therefore, in simulation studies described here, we extend the idealized model by adding noise to the decision variable Φ*x* as follows:

2 $$y=\operatorname{sign}\left(\Phi x+\varepsilon \right),$$

where *ε* is additive noise. This model of noise is consistent with classic results (e.g., Burgess & Colbourne, 1988) showing that internal observer noise can be modeled as a perturbation to the decision variable, prior to the subject response being rendered.

### Reverse correlation reconstruction

The subject response model is inverted in conventional reconstruction of the latent representation (e.g., Abbey & Eckstein, 2002; Gosselin & Schyns, 2003), in a method closely related to regression of the responses against the stimuli, and similar to that used in the spike-triggered averaging (STA) approach to uncovering neural tuning (e.g., receptive fields):

3 $$\hat{x}={\left({\Phi}^{\mathrm{T}}\Phi \right)}^{-1}{\Phi}^{\mathrm{T}}y.$$

This equation is commonly simplified, using the knowledge that the stimuli in typical reverse correlation experiments are uncorrelated, making it unnecessary to calculate the full sum of squares and cross products matrix, and instead simply normalize by the number of responses gathered:

4 $$\hat{x}={\mathrm{m}}^{-1}{\Phi}_y,$$

which amounts to an averaging procedure, whereby the mean of stimuli eliciting a "no" response is subtracted from the mean of stimuli eliciting a "yes" response.

### Compressive sensing reconstruction

Compressive sensing uses a closely related model of subject behavior as above but assumes that the latent representation is sparse in some basis domain (i.e., it is compressible). The objective of compressive sensing is to estimate the sparse representation directly, which will be expected to be more efficient than reconstructing the full representation itself. Given this assumption, the model of subject behavior becomes:

5 $$y=\operatorname{sign}\left(\Phi \Psi s\right),$$

where *s* is the sparse representation, and Ψ is a matrix of orthonormal basis functions, specifically the 2D discrete cosine basis. It is helpful to calculate the similarity between the stimuli and the basis functions via matrix multiplication, producing a matrix of such similarity (i.e., inner product) values, Θ, which then leads to:

6 $$y=\operatorname{sign}\left(\Theta s\right).$$

This I-by-m matrix is sometimes known as the "compressive sensing matrix", which can be used as the basis for estimating s. The process of estimation has been an active area of research since the beginning of compressive sensing research, and many algorithms have been proposed, depending on assumptions about the nature of the responses, the error criteria to be optimized, and the method of optimizing a given criterion. In the present paper, we highlight the method of Zhang et al. (2014), which is intended to operate on binary, yes/no responses, and features a simple calculation and closed-form solution for finding an estimate of *s*:

7 $$\hat{s}={\upzeta}^{-1}\mathrm{P}\left({\mathrm{m}}^{-1}{\Theta}^{\mathrm{T}}y\right),$$

where P(.) is a soft thresholding operation that zeros all but the γ elements of the vector with the largest absolute magnitude, and the normalizing factor ζ = ||P(m^-1^Θ^T^*y*)||. The variable γ is the degree of sparsity, such that the solution will be γ-sparse.

As mentioned above, conventional reverse correlation can be viewed as taking the sum of all the “yes” stimuli and subtracting from that the sum of all the “no” stimuli. The compressive sensing approach described here performs a similar difference-of-sums, but rather than doing so over the stimuli, it performs the difference-of-sums over the stimuli transformed using the basis vectors. For instance, if the basis vectors are cosines of different frequencies, then the difference-of-sum will be done over the DCT (discrete cosine transform) coefficients representing the stimuli. In compressive sensing, the elements of the resulting vector are then soft-thresholded to promote sparsity, such that those with a small absolute magnitude are set to zero. In both methods, the elements of the vectors are then normalized.

Once *s* has been estimated, it can be used in conjunction with Ψ to reconstruct the latent representation *x* using the known basis functions:

8 $$\hat{x}=\Psi \hat{s}.$$

The signum function is used to model the transformation of stimulus-representation comparisons, the result of which can be a continuous value, into this numerical response code. Issues surrounding quantization of the responses – with reduction to binary, yes/no values being the most extreme example – are of theoretical and practical interest. Classical compressive sensing assumes the measurement of continuous response values, although compressive sensing over binary responses (termed "one-bit compressive sensing" or "binary compressive sensing") has been studied extensively, leading to the reconstruction process incorporated into the present framework (Zhang et al., 2014) and several other techniques (Jacques et al., 2013). One-bit compressive sensing continues to be an active area of research (e.g., Shen, 2020). In the case of experimental paradigms where continuous or mildly quantized responses can feasibly be collected from subjects, classical compressive reconstruction techniques (e.g., Blumensath & Davies, 2008) may be appropriate, and perhaps superior, to employ. It seems that eliciting yes/no responses from subjects in the context of perceptual experiments is likely to remain the standard method because it is the most straightforward for subjects and experimenters to implement.

Note that the subject response model assumed within a compressive sensing context (Eq. 5) is closely related to sparse models developed by Mineault (2009). Indeed, when looking to solve the problem of finding an optimal value for *s*, Mineault reached for an optimization algorithm developed with compressive sensing applications in mind – specifically, the fixed-point continuation algorithm proposed by Hale et al. (2007). However, explicit identification of that problem as one that can be solved using compressive sensing (not provided by Mineault) allows for a deeper analysis of which techniques available in the sizeable compressive sensing literature are most appropriate for the problem at hand.

The focus on binary, yes/no responses in a reverse correlation context points to similarities with 1-bit compressive sensing, which also focuses on binary responses and has been studied extensively beginning at least with the work of Bounfounos & Baraniuk (2008). Furthermore, the fact that human subjects’ responses are often noisy points to the need for robust compressive sensing that looks at accurate estimation despite the presence of noise. A landmark result in this area was due to Plan & Vershynin (2012), who cast one-bit, robust compressive sensing as the following optimization problem:

9 $$\max\ \mathrm{\it s}\ \mathrm{of}\ {\mathrm{\it s}}^{\mathrm{T}}\Theta \mathrm{\it y}\kern0.5em \mathrm{s}.\mathrm{t}.\kern0.5em {\left\Vert \mathrm{\it s}\right\Vert}_2\le 1,{\left\Vert \mathrm{\it s}\right\Vert}_1\le \mathrm{\it s}\mathrm{qrt}\left(\uplambda \right)$$

where λ is a threshold that determines the degree of sparsity of the solution. Inspired by this result, Zhang (2014) was able to cast 1-bit, robust compressive sensing as the following optimization problem:

10 $$\min\ \mathrm{\it s}\ {\left\Vert \mathrm{\it s}\right\Vert}_2\le 1,{\mathrm{m}}^{-1}{\mathrm{\it s}}^{\mathrm{T}}\Theta \mathrm{\it y}+\uplambda {\left\Vert \mathrm{\it s}\right\Vert}_1$$

and show that this optimization problem has an analytical solution, which is Eq. (7). In the present work, the value of λ is always determined such that the solution will be γ-sparse.

Functions implemented in MATLAB code for compressive sensing (Eq. 7), reverse correlation (Eq. 4), and simulated subject response generation (Eq. 1) are available at https://github.com/alammert/compressive-sensing, along with a MATLAB script constituting a working example exemplifying their use.

### Sparse generalized linear model

To compare compressive sensing to an established sparse method for generating reverse correlation reconstructions (Murray, 2011), the generalized linear model (GLM) with sparse priors described by Mineault (2009) was used to generate reconstructions of the latent representations. The method works by finding the maximum a posteriori estimate of the representation using a sophisticated optimization algorithm. The code to do this was downloaded from the online repository (http://packlab.mcgill.ca/publications.html, accessed May 24, 2022).

The method employs a fixed-point iteration algorithm due to Hale et al. (2007) to fit a GLM in accordance with:

11 $$\text{argmin}_s ={L}_b+{\uplambda} {\left\Vert {s}\right\Vert}_1,$$

where *L*_*b*_ is the negative log-likelihood function for the model of subject behavior:

12 $$\kern0.5em {L}_b=-{\sum}_{\mathrm{i}=1}^{\mathrm{n}}\log\ f\left({y}_i{\left(\Phi \Psi \it s+ Uu\right)}_{\mathrm{i}}\right),$$

with auxiliary variables *U* and corresponding weights *u*, explained in Mineault (2009), and where *f*(∙) is the logistic function. Note that the matrix of basis functions, Ψ, used with the GLM method was the 2D discrete cosine basis, consistent with the proposed compressive sensing method.

### Simulating Gosselin & Schyns (2003)

The latent representation, *x*, was generated by recreating the “superstitious” perception of “S” for subject NL from Gosselin & Schyns (2003) original work as a *template* for simulating NL’s responses. The “S” was recreated by horizontally scaling a lowercase Verdana “S”, as described in the original manuscript, and resizing the letter to 50 × 50 pixels using MATLAB (R2021a, The MathWorks, Inc.). This “S” was then converted to grayscale pixel intensity values scaled between 0 and 1. Random stimuli (Φ) were generated by randomly drawing values 0 and 1 with equal probability. Subject responses to the stimuli were simulated as described in Eq. (2), using the template “S” as *x*, and with *ε ~ N(0,σ)*, a Normal distribution with mean zero and variance σ.

Simulations of Gosselin & Schyns (2003) were conducted with 1250, 2500, 5000, 10,000, and 20,000 samples. For each number of samples, ten simulations obtained representation estimates using conventional reverse correlation, another ten used compressive sensing, and another ten used the sparse GLM from Mineault et al. (2009). For all simulations at a given number of samples, the mean reconstruction quality (see below) and 95% confidence intervals were calculated separately for the reverse correlation, compressive sensing, and GLM estimates. Simulations of noisy subject responses were also conducted, following the model described in Eq. (2). Ten additional simulations were conducted at each number of samples with this added noise (ten simulations at *σ* = 25 and ten at *σ* = 50) for each of reverse correlation, sparse GLM, and compressive sensing. The variance value σ can be thought of as the percent of incorrect responses generated by the simulated observer. In each simulation condition, the value of γ was determined by examining the prediction accuracy (see “Assessing Reconstruction Quality”, below) across a range of γ values on held-out data, specifically novel data sets generated using Eq. (2). Eight such novel data sets were generated in each condition, and the mean prediction accuracy was calculated. The values of γ considered comprised {2, 4, 8, 16, 32 ,64, 128, 256, 512, 1024, 2048}. It was found that γ = 64 provided the best mean accuracy in all simulation conditions. Similar experiments were performed with a letter “K”, the phoneme /i/ (as presented by Mesgarani et al. (2009), and a dot/impulse (Fig. S1), as detailed in supplemental material.

### Compressive sensing on human data

Human data from Smith et al. (2012) was obtained for use in this paper. In the original paper, subjects (*n* = 5) were presented with binary noise visual stimuli and were asked to identify if they perceived a face in the image; unbeknownst to the subjects, none of the images contained a face. Reverse correlation was used to reconstruct each subject’s internal representation of a face. Here, conventional reverse correlation, reverse correlation with compressive sensing, and estimation using a sparse GLM were performed on the stimulus-response pairs for each subject. For reverse correlation, representation reconstructions were generated by multiplying the matrix of stimuli by the vector of responses. For compressive sensing, *ŝ* was calculated and multiplied by Ψ to reconstruct the representation. For the sparse GLM method, a GLM was fit as described in (Mineault et al., 2009) and was used to estimate the representation. Response values of 0 were mapped to – 1 to be compatible with the original code.

The parameter γ was determined through a ten-fold cross-validation for each subject individually. For a subject, the matrix of orthonormal basis functions, Ψ, was generated, and Ψ was regressed against 8/10 of the stimuli to produce Φ. Φ, γ and 8/10 of the subject responses were used to produce *s*, the sparse weights, as in Eq. (7). The weights were then regressed against Ψ to generate a compressive sensing estimate of the subject’s internal representation of a face, as in Eq. (8). The resulting reconstruction then served as a template for the simulated response generating procedure, whereby another 1/10 of the stimuli were regressed against the estimated template to generate estimated subject responses. The balanced accuracy between these estimated responses and the actual responses for these 1/10 of the stimuli was calculated. This procedure was done with γ in {2, 4, 8, 16, 32 ,64, 128, 256, 512, 1024}. The value of γ that produced the highest accuracy was used to generate a reconstruction with the remaining 1/10 of the data, and the accuracy of this reconstruction (the “test accuracy”) was calculated. This procedure was repeated using each tenth of the stimulus-response pairs to compute the test accuracy, and the value of γ that most frequently produced the highest test accuracy was selected as the appropriate gamma for that subject. This procedure was repeated individually for each of the five subjects, and the selected γ values for S1-S5 were 64, 16, 64, 32, and 64, respectively.

### Assessing reconstruction quality

Two measures of reconstruction quality were employed: (1) similarity of the reconstruction to the template by direct comparison (for simulated data only), and (2) accuracy obtained from using the reconstruction to predict subject responses. The first measure more specifically used Pearson’s *r* to quantify similarity between reconstructions and the corresponding templates the responses were generated from. The second measure relies on predicted subject responses that can be made by applying Eq. (2) to held-out or novel data using reconstructions in place of *x*. Reconstruction quality can then be calculated and expressed as balanced accuracy over such predictions. In assessing prediction accuracy on human data, the second measure was conducted using within-subject five-fold cross validations separately on estimates obtained from each reconstruction method (detailed below). In assessing prediction accuracy on simulated data, a novel data set was generated for the assessment using Eq. (2), which eliminated the need for implementing a cross-validation scheme.

Both measures were used to assess reconstruction quality in simulation studies, while only the second measure was used to assess reconstruction quality from human data. In simulation studies, the quality of reconstructions can be determined directly by comparing reconstructions against the template because the entire response process is observable, whereas the template/representation is not directly observable in the context of human data.

To assess the quality of the reconstruction estimation procedure for the human data, stimuli and responses from each of the five subjects (S1-S5) from Smith et al. (2012) were used for a fivefold cross-validation procedure. For a subject, 4/5 of the stimuli and the corresponding responses were used to generate a reconstruction using Eq. (4). That reconstruction then served as a template for the simulated response generating procedure with the remaining 1/5 of the stimuli, whereby the remaining 1/5 of the stimuli were regressed (compared) against the template reconstructed from 4/5 of the stimulus-response pairs to generate estimated subject responses, as in Eq. (2). This procedure was repeated for each fifth of the stimulus-response pairs for a subject. The simulated subject responses for all cross-validations were compared to the actual human responses, and balanced accuracy was calculated. This cross-validation was conducted independently on the data for each of the five human subjects.

A similar cross-validation was performed on the human data to assess the compressive sensing simulation model. The matrix of orthonormal basis functions, Ψ, was generated for each subject, and Ψ was regressed against 4/5 of the stimuli to produce Φ. The determined γ value for the subject, Φ, and 4/5 of the subject responses were used to produce *s*, the sparse weights, as in Eq. (7). The weights were then regressed against Ψ to generate a compressive sensing estimate of the subject’s internal representation of a face, as in Eq. (8). The resulting reconstruction then served as a template for the simulated response generating procedure, whereby the remaining 1/5 of the stimuli were regressed against the estimated template to generate estimated subject responses. This procedure was repeated for each fifth of the stimulus-responses for a subject. The simulated estimated subject responses for all cross-validations were compared to the actual human subject responses, and balanced accuracy was calculated. The cross-validation was conducted independently on the data for each of the five human subjects.

Cross-validation was again performed on the data to assess the simulation with Mineault’s GLM. The GLM was fit with 4/5 of the stimuli and 4/5 of the subject responses, and the fit GLM’s output was the estimate of the subject’s internal representation of a face. That reconstruction then served as a template for the simulated response generating procedure. The remaining 1/5 of the stimuli were regressed against the estimated template to generate estimated subject responses. This procedure was repeated for each fifth of the stimulus-responses for a subject. The produced estimated subject responses for all cross-validations were compared to the actual human subject responses, and balanced accuracy was calculated. The cross-validation was conducted independently on data for each of the five human subjects.

Quality of reconstructions from compressive sensing was compared to that obtained from conventional reverse correlation and the sparse GLM by performing a hypothesis test on the balanced accuracies produced by the aforementioned cross-validation. A paired *t* test was performed across subjects with the null hypothesis that the mean difference in balanced accuracies obtained from compressive sensing versus those obtained from reverse correlation or the Sparse GLM was zero. A level of α = 0.05 was used as the significance threshold.

We note that methods for assessing reconstruction quality in the case of human data have been the subject of discussion in the literature (Murray, 2011). Here, we follow the approach suggested by Neri & Levi (2006), that validates estimates by considering their ability to predict subject responses on a trial-by-trial basis using the same subject response model discussed in Eq. (2). Prior efforts following this approach have focused on showing that predicted responses using the estimated representation were close to a theoretical upper bound for accuracy (akin to goodness of fit assessment), suggesting that the representation is a valid encapsulation of subject behavior given the response model. Prediction accuracy in such approaches is bounded by any noise in the subject response process (Murray et al., 2005), and so determining the overall validity of the estimate requires an estimate of the level of subject response noise. Here, the goal is rather simpler: to show that predicted responses using the compressive sensing-derived estimate are more accurate than predicted responses using other methods of reconstruction.

## Results

### Compressive sensing improves reconstruction accuracy in simulated data

**Fig. 3:**
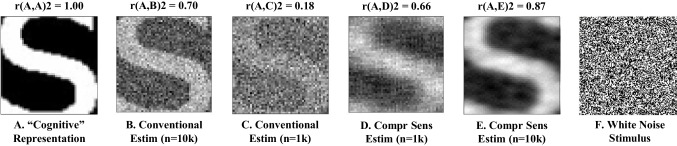
Comparison of conventional regression-based estimation and compressive sensing estimation**.** The template image **(A)** of the “S” from Gosselin & Schyns (2003) is estimated in **(B–E)**. An example noise stimulus is shown in **(F)**. The method of estimation and number of samples used (n) is indicated below the image. The correlation coefficient (*r*^2^) between the template and the estimate is shown above the image, indicating estimation quality. In the simulation, compressive sensing provides equivalent accuracy as the conventional approach with only 10% as many trials

To directly assess the applicability of compressive sensing to recovering cognitive representations, we compare the results of simulations of Gosselin & Schyns’ original study on representations of “S” (Fig. 3) using both conventional reverse correlation (Gosselin & Schyns, 2003) and compressive sensing approaches. We used an image of the letter “S” as the target cognitive representation for reconstruction (Fig. 3A). Specifically, the reshaped, vectorized “S” image was used as the representation vector, *x* (Fig. 2), the basis for generating simulated subject responses in light of random stimuli contained in the stimulus matrix, Φ. The elements of Φ were generated by randomly sampling from a uniform distribution over the interval 0 and 1 and rounding those values to the nearest integer. A total of 10,000 subject responses were simulated using this procedure, representing the subject responses in Gosselin & Schyns (2003). In the simulations, as in most cognitive experiments, we assume binary responses.

The simulated subject responses were used as the basis for reconstructing the latent representation, *x*, using both conventional regression-based estimation and compressive sensing-based estimation. Reconstructions were generated using both the full set of 10,000 stimulus-response pairs and using a limited subset of a randomly selected 10% of pairs (1000). Quality of a given reconstructed representation was assessed using two-dimensional correlation coefficient (*r*^2^) between the target representation and the reconstructed estimate. Results (Fig. 3) indicate that the accuracy of the reconstructed representation obtained using 10% of the samples via compressive sensing is effectively equivalent to that obtained using the full cohort of samples via conventional reconstruction, implying a 90% improvement in sampling efficiency. When allowed to operate on the full complement of samples, compressive sensing additionally shows improved accuracy over conventional reconstruction of approximately 15%. Similar improvements were shown on other types of naturalistic stimuli (Figs. S2–S3).

### Compressive sensing outperforms reverse correlation across sample sizes and levels of noise in participant responses

**Fig. 4:**
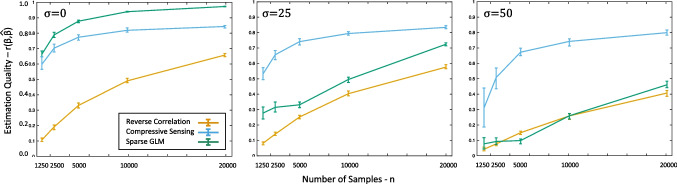
Estimation quality as a function of sample size. Estimation quality (mean and 95% CI) obtained from reverse correlation, compressive sensing, and the sparse GLM from (Mineault et al., 2009) in our stimulation study shown across a range of sample sizes. Estimation quality is shown separately for various levels of noise in the subject responses, including no noise (σ = 0), moderate noise (σ = 25), and heavy noise (σ = 50). At all sample sizes and levels of noise, compressive sensing provides a much more accurate reconstruction than conventional reverse correlation, and the performance of the GLM diminishes when noise is added to the responses

**Fig. 5:**
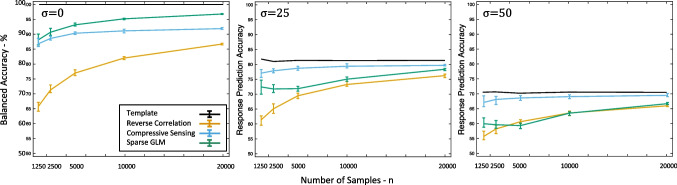
Response prediction accuracy as a function of sample size. Accuracies (mean and 95% CI) in predicting responses using reconstructions obtained from the template, reverse correlation, compressive sensing, and the sparse GLM from (Mineault et al., 2009) in our stimulation study shown across a range of sample sizes. Estimation quality is shown separately for various levels of noise in the subject responses, including no noise (σ = 0), moderate noise (σ = 25), and heavy noise (σ = 50). At all sample sizes and levels of noise, compressive sensing provides a much more accurate reconstruction than conventional reverse correlation, and the performance of the GLM diminishes when noise is added to the responses. Correlation of the noisy template with the template is shown as the *solid black line*

An additional simulation study was conducted to provide a more complete view of the performance of compressive sensing as a function of the number of samples. This study followed Gosselin & Schyns’ (2003) letter "S" example, as above, varying the number of stimulus-response pairs between 1250 and 20,000. A total of 30 simulations were conducted with each number of pairs, with ten obtaining estimates using conventional reverse correlation, another ten using compressive sensing, and another ten using the sparse GLM. For all simulations at a given value of *n*, the mean estimation quality (*r*^2^) and 95% confidence intervals were calculated separately for reverse correlation, compressive sensing and sparse GLM estimates (Fig. 4), and the prediction accuracy and 95% confidence intervals were also calculated in the same conditions (Fig. 5).

The simulations described above assume an ideal observer – essentially a participant that does not make mistakes. However, human subjects are not ideal observers but rather will sometimes provide responses that are incorrect with respect to the task (i.e., responding "no" to a stimulus that well-matches the representation, or responding "yes" to a stimulus that doesn't). Importantly, prior results have demonstrated both theoretically and empirically that compressive sensing is robust to such noise (Zhang et al., 2014; Donoho, 2006). In order to verify those prior results and reproduce them in the compressive sensing context, we conducted a series of additional simulations that relaxed the assumption of an ideal observer. Specifically, “noisy” subject responses were modeled in accordance with Eq. (2). Levels of error considered, in addition to *σ* = 0, included *σ* = 25 and *σ* = 50. Results of these simulations are shown in Figs. 4 and 5. As expected, less accurate responses (higher *σ* values) produced lower estimation qualities and prediction accuracies across all sizes of stimulus-response pairs. Critically, however, compressive sensing continued to outperform conventional reverse correlation in all cases. Similar improvements were shown with compressive sensing when the stimulus was a different letter (Fig. S2) or a phoneme (Fig. S3), although the sparse GLM outperforms compressive sensing when the stimulus was a dot/impulse (Fig. S4).

### Compressive sensing improves reverse correlation on data from human participants

**Fig. 6:**
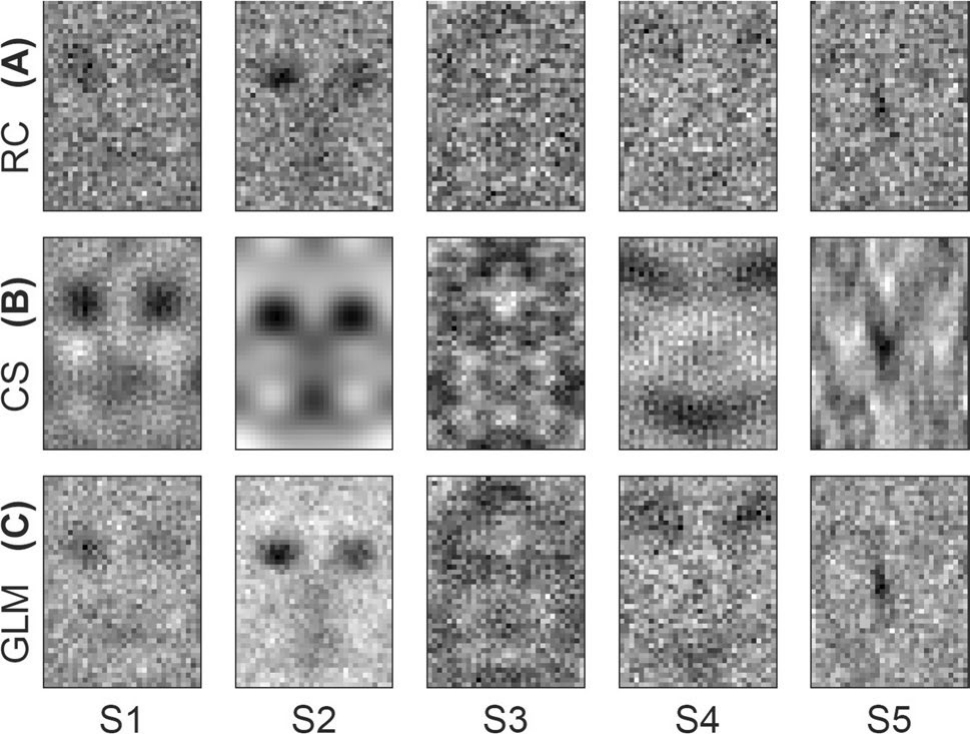
Comparison of conventional regression-based estimation and compressive sensing estimation of faces from human response data. **(A)** The reconstructions created with reverse correlation from the data collected by Smith et al., (2012). **(B)** The compressive sensing reconstructions. **(C)** Color-adjusted reconstructions generated from the sparse generalized linear model described by Mineault (2009) for subjects S1–S5. Structural features identified in (A), such as a nose, mouth, and chin outline on S1, S2, and S3, are more noticeable in (B). GLM reconstructions in (C) have been brightened for clearer viewing (see Methods). Note that the faces for S1 and S2 appear switched compared to the 2012 paper, but they are named as labeled in the provided data

Human response data is the gold standard when proposing a technique intended to improve the processing of human subject responses. To this end, we reanalyzed human response data from Smith et al. (2012) in which reverse correlation was used to reconstruct internal templates of faces. In that study, five subjects were each shown approximately 10,500 binary noise images and asked to identify those stimuli with a face in them. Here, reverse correlation, sparse GLM estimation, and compressive sensing were all performed on the actual data from Smith et al. (2012) to generate reconstructions. The reconstructions using reverse correlation, essentially a recapitulation of the original results, are shown in Fig. 6A. As may be expected in a sparse image, the compressive sensing reconstructions have a smoother, more face-like appearance (Fig. 6B). Furthermore, Smith et al. (2012) identified several facial structures in their reconstructions (e.g., a hairstyle on subject S3 and a nose, mouth, and chin outline for S1, S2, and S3), and these are more clearly observable in the reconstructions using compressive sensing. The reconstructions presented here are notably symmetric and lack high frequency variation. These properties are not enforced by the compressive sensing method. Rather, faces themselves are largely symmetric, and these reconstruction properties are driven by the data.

**Fig. 7:**
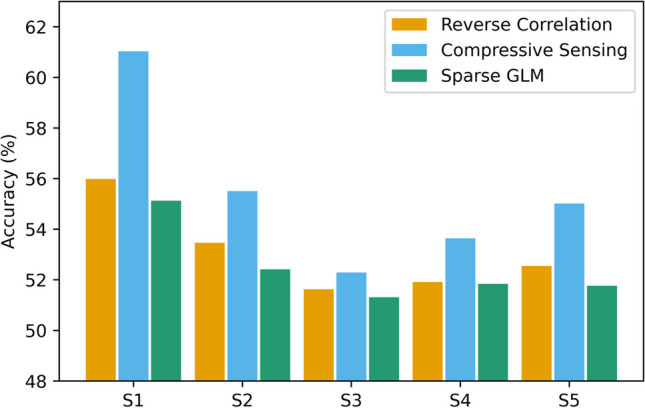
Balanced accuracies of cross-validation for five human subjects. Human subject response data (Smith et al., 2012) was used to generate a reconstruction with reverse correlation, compressive sensing, or the sparse GLM technique from (Mineault et al., 2009), and simulated responses to stimuli were generated using the reconstruction as a template. A fivefold cross-validation was done to compare the subject responses to the simulated responses for each subject. For all subjects, compressive sensing provides a more accurate estimate of responses than either conventional reverse correlation or the GLM method

For all subjects, compressive sensing yielded a higher balanced accuracy than did conventional reverse correlation or the sparse GLM (Fig. 7). Most markedly, using compressive sensing over reverse correlation increased the accuracy for subject S1 from 56 to 61%, a nearly 9% upward change. A paired *t* test, performed across subjects under the null hypothesis that the mean difference in balanced accuracies obtained from compressive sensing versus those obtained from reverse correlation was zero, was found to be statistically significant (*p* = 0.031). A paired *t* test, performed across subjects under the null hypothesis that the mean difference in balanced accuracies obtained from compressive sensing versus those obtained from the sparse GLM was zero, was found to be statistically significant (*p* = 0.023).

## Discussion and conclusion

Reverse correlation is a technique commonly used in neuroscience and psychology research which requires gathering many stimulus-response pairs. When used in cognitive studies to infer internal representations of target stimuli, the high number of trials needed to produce an adequate reconstruction can make a study burdensome or even infeasible. Despite this inefficiency, reverse correlation is widely used to uncover lower-level perceptual and neural representations and has had impactful initial success when applied to higher-level cognitive representations. Here, we propose an improvement to conventional reverse correlation by coupling it with compressive sensing, an advanced signal processing technique designed to enhance sampling efficiency.

Our results suggest that compressive sensing can reduce the number of trials required for accurate estimation of cognitive representations using reverse correlation by up to 90% without any loss of accuracy. Because estimating representations with traditional reverse correlation typically requires the collection of several thousands of stimulus response pairs, very few participants are examined in any given study, which limits the possible analyses and inferences about potential universal aspects of human cognitive representation, as well as examination of individual differences. The 90% reduction in required trials demonstrated here opens the door to conducting more studies with more participants. Cognitive studies that would presently take weeks to complete could instead be performed in one sitting, and those studies that would otherwise impose limiting constraints on either the stimuli or the reconstructions (e.g., Moon et al., 2020) could be conducted with assumptions only about sparsity; this would lead to clearer, less biased representations. Given the central importance of cognitive representations in mediating human perceptual experience and behavior, this presents a substantial increase for reverse correlation’s potential for wide-spread use in the psychological and cognitive sciences.

Alternatively, the compressive sensing approach may be used to generate more accurate reconstructions for a given number of trials, as was done here with representations of faces. The same number of stimulus-response pairs generated a clearer face than in the original work (Smith et al., 2012), and using compressive sensing created reconstructions that elicited more accurate simulated responses than reverse correlation or the GLM in the cross-validation (Fig. 7). This same improvement in reconstruction quality could be seen in other cases where a full complement of trials is possible to collect, or even retrospectively in the context of already-collected data sets.

The simulations reconstructing “S” showcase compressive sensing’s utility for analyzing novel data, yet compressive sensing is not limited to use solely in new studies. Rather, reanalysis of human response data from (Smith et al., 2012) shows that compressive sensing can also be retrospectively applied to existent data to improve results. Real human subject responses are the gold standard data when proposing a technique intended to improve the processing of human subject responses. However, whereas the human subject responses demonstrate compressive sensing’s utility on real data, the simulations also used in this work offer the benefit of being an entirely observable process, including the access to underlying representations that the responses are based off of. This means that the quality of the reconstruction can be assessed directly by comparing the representation to the reconstruction. The subject response model used in simulation here is consistent with that assumed in other applications of reverse correlation (Ahumada, 2002). What’s more, this work takes the additional step of modeling subject noise in the responses (Fig. 4).

Both compressive sensing and the sparse GLM greatly outperformed traditional reverse correlation in simulations without added noise. Under noiseless conditions, the performance of sparse GLM and compressive sensing is comparable when the number of trials is small, and the Sparse GLM performs somewhat better than compressive sensing when the number of trials is large (Fig. 4). Critically, any advantage of the GLM approach was not present when noise was added to simulate subject responses that are more realistic (i.e., imperfect). Prior attempts to improve the efficiency of reverse correlation using GLMs have shown that the benefit over traditional linear reverse correlation diminishes as more noise is added (Mineault et al., 2009; Abbey & Eckstein, 2001; Knoblauch & Maloney, 2008), and the present work further demonstrates that. When the noise level was at 20%, the GLM performed similarly to reverse correlation at all values of *n*. These results were further supported when using real human data, which is inherently noisy; compressive sensing had a higher balanced accuracy than the GLM for all five subjects, and the GLM performed similarly to the standard reverse correlation approach. Furthermore, the compressive sensing procedure used here (adapted from Zhang et al., 2014) was several orders of magnitude faster than the optimization method of the GLM from (Mineault et al., 2009).

Given earlier discussion regarding 1-bit, robust compressive sensing being highly appropriate in the present context, we can speculate that the poor performance of Mineault’s method compared to the presently employed method of Zhang (2014) may be due to either (a) reliance on an iterative, as opposed to analytical, solution, which may result in failure to minimize the objective function with sufficient precision, or (b) failure to effectively deal with the high levels of noise typical of real human responses.

Despite the apparent advantages of taking a compressive sensing approach to reverse correlation, careful consideration should nevertheless be given for specific applications regarding whether the target representations can reasonably be expected to be sparse. Even though the assumption of sparsity, which is so critical to the successfully application of compressive sensing, seems well justified for images and visual representations, the assumption of sparsity will be expected to bias results toward sparse estimates even when the underlying representation is non-sparse.

Compressive sensing holds promise for drastically improving the efficiency and accuracy of analysis in reverse correlation beyond cognitive science, in domains including a broad range of electrophysiology paradigms (e.g., De Boer & Kuyper, 1968; Ringach & Shapley, 2004; Stark, 1968), calcium imaging (Ramdya et al., 2006), auditory system function (Ahumada and Lovell, 1971), and gene expression (Lin et al., 2015). Given that reverse correlation responses can be continuous (e.g., neural firing rates; Ringach & Shapley, 2004), ordinal (e.g., similarity scores; Zymnis et al., 2009), or binary (e.g., yes/no; Zhang et al., 2014; Boufounos & Baraniuk, 2008; Jacques et al., 2013), compressive sensing has the potential to be widely utilized in areas of science beyond where reverse correlation is currently in common use.

### Supplementary information

ESM 1 (DOCX 1488 kb)
