# Supplementary material for: A compressive sensing approach for inferring cognitive representations with reverse correlation
Source: Behav Res Methods. 2023 Dec 4;56(4):3606–18. doi: 10.3758/s13428-023-02281-4 (PMC11133035; doi:10.3758/s13428-023-02281-4)
Supplement: Supplementary file 1 — (DOCX 1488 kb) [file 13428_2023_2281_MOESM1_ESM.docx]

Supplement to “A Compressive Sensing Approach for Inferring Cognitive Representations

with Reverse Correlation”

Benjamin W. Roop^1^, Benjamin Parrell^2^, Adam C. Lammert^1,3^

^1^ Program of Neuroscience, Worcester Polytechnic Institute

^2^ Department of Communication Sciences and Disorders, University of Wisconsin-Madison

^3^ Biomedical Engineering Department, Worcester Polytechnic Institute

Supplemental simulation studies were performed that were identical to those described in the main paper, but substituting alternatives to the letter “S” template. The purpose of these simulation studies was to give an indication of how the estimation quality and response prediction accuracy results vary as a function of the template used.

**Figure S1: Templates used in the supplemental simulation studies.** Templates used in supplemental simulation studies: letter “K” (A), phoneme /i/ (B), and a dot/impulse (C).

The templates used in these supplemental simulations studies are shown in Fig S1, and comprise (A) an uppercase Verdana “K”, (B), an adaptation of the spectrogram for the phoneme /i/, as in “heed”, presented by Mesgarani et al. (2009), and (C) a dot/impulse image. The letter “K” was chosen as a complement to the letter “S”, being a letter that, unlike “S”, contains straight edges and lacking symmetry. The phoneme /i/ was chosen as an example of a relevant image from a non-visual domain that has different shape and smoothness properties to the letter examples. The dot/impulse image was chosen as an example of a non-natural image that cannot be represented sparsely in standard basis sets.

**Figure S2: Estimation quality and response prediction accuracy as a function of sample size – letter “K”.** Estimation quality (mean and 80% CI) obtained from reverse correlation, compressive sensing, and the sparse GLM from (Mineault et al., 2009) in a supplemental simulation study identical to that described in the present paper, but using the letter “K” (Fig S1a) as a template.

The parameter $\gamma$ was determined using cross-validation, as in the experiments with letter “S”. A value of $\gamma=64$ was selected by the cross-validation procedure in all situations involving letter “K” and phoneme /i/. A much higher value of $\gamma=2048$ was selected by the cross-validation procedure in situations involving the dot/impulse image, likely due to the fact that this image cannot be represented sparsely in the cosine basis used here.

Estimation quality and response prediction accuracy for letter “K”, the phoneme /i/ and the dot/impulse image are shown in Figs S2, S3 and S4, respectively. The results for the letter “K” and the phoneme /i/ are substantially similar to those presented in the main paper for the letter “S”, indicating that those results may not vary substantially with the target representation, provided that the representation of interest is a natural signal. The results for the dot/impulse image indicate that compressive sensing may struggle to estimate non-natural representations that cannot be represented sparsely in the chosen basis, as compressive sensing performed comparably with conventional reverse correlation in that case. It appears that Mineault’s (2009) method may be a better choice for such representations. However, it is notable that estimation quality for the dot/impulse image becomes considerably poorer for all methods with any increase in subject response noise.

**Figure S3: Estimation quality and response prediction accuracy as a function of sample size – phoneme /i/.** Estimation quality (mean and 80% CI) obtained from reverse correlation, compressive sensing, and the sparse GLM from (Mineault et al., 2009) in a supplemental simulation study identical to that described in the present paper, but using the phoneme /i/ (Fig S1a) as a template.

**Figure S4: Estimation quality and response prediction accuracy as a function of sample size – dot/impulse image.** Estimation quality (mean and 80% CI) obtained from reverse correlation, compressive sensing, and the sparse GLM from (Mineault et al., 2009) in a supplemental simulation study identical to that described in the present paper, but using a dot/impulse image (Fig S1a) as a template.
